# Integrating gestational diabetes and type 2 diabetes care into primary health care: Lessons from prevention of mother-to-child transmission of HIV in South Africa - A mixed methods study

**DOI:** 10.1371/journal.pone.0245229

**Published:** 2021-01-22

**Authors:** Jean Claude Mutabazi, Pascal Roland Enok Bonong, Helen Trottier, Lisa Jayne Ware, Shane A. Norris, Katherine Murphy, Naomi Levitt, Christina Zarowsky

**Affiliations:** 1 Département de médecine sociale et préventive, École de Santé Publique, Université de Montréal, Montréal, QC, Canada; 2 Centre de recherche en santé publique (CReSP), Université de Montréal et CIUSSS du Centre-Sud-de-l’Île-de-Montréal, Montréal, Canada; 3 Centre de Recherche du Centre Hospitalier de l’Universitaire Sainte Justine, Montréal, QC, Canada; 4 SAMRC Developmental Pathways for Health Research Unit, Department of Paediatrics, School of Clinical Medicine, University of the Witwatersrand, Johannesburg, South Africa; 5 Division of Endocrinology, Department of Medicine, Faculty of Health Science, University of Cape Town, Chronic Disease Initiative for Africa, Cape Town, Western Cape, South Africa; 6 School of Public Health, University of the Western Cape, Bellville, South Africa; 1. IRCCS Neuromed 2. Doctors with Africa CUAMM, ITALY

## Abstract

**Background:**

Implementation of the programmes for the Prevention of Mother to Child Transmission (PMTCT) of Human Immunodeficiency Virus (HIV) into antenatal care over the last three decades could inform implementation of interventions for other health challenges such as gestational diabetes mellitus (GDM). This study assessed PMTCT outcomes, and how GDM screening, care, and type 2 diabetes (T2DM) prevention were integrated into PMTCT in Western Cape (WC), South Africa.

**Methods:**

A convergent mixed methods and triangulation design were used. Content and thematic analysis of PMTCT-related policy documents and of 30 semi-structured interviews with HIV/PMTCT experts, health care workers and women under PMTC diagnosed with GDM complement quantitative longitudinal analysis of PMTCT implementation indicators across the WC for 2012–2017.

**Results:**

Provincial PMTCT and Post Natal Care (PNC) documents emphasized the importance of PMTCT, but GDM screening and T2DM prevention were not covered. Data on women with both HIV and GDM were not available and GDM screening was not integrated into PMTCT. Women who attended HIV counselling and testing annually increased at 17.8% (95% CI: 12.9% - 22.0%), while women who delivered under PMTCT increased at 3.1% (95% CI: 0.6% - 5.9%) annually in the WC. All 30 respondents favour integrating GDM screening and T2DM prevention initiatives into PMTCT.

**Conclusion:**

PMTCT programmes have not yet integrated GDM care. However, Western Cape PMTCT integration experience suggests that antenatal GDM screening and post-partum initiatives for preventing or delaying T2DM can be successfully integrated into PMTCT and primary care.

## Background

South Africa (SA) has extensive programmes for Human Immunodeficiency Virus (HIV) counselling and testing (HCT) and lifelong antiretroviral therapy (ART) aimed at achieving national and global HIV control targets [1, 2]. These include integrated primary health care (PHC) based post-partum follow-up for both mothers and their babies, who undergo early diagnosis using HIV polymerase chain reaction (PCR) testing [1, 3]. With the implementation of Prevention of Mother-to-Child Transmission (PMTCT) of HIV, and its subsequent integration into existing primary health care services, coverage has increased over time, especially in the wake of the initiation of lifelong ART immediately after diagnosis, regardless of CD4 count or clinical staging [4–6]. This approach, known as Option B+ for PMTCT, was included in national policies and implemented in more than 95% of countries worldwide, resulting in the considerable reduction of infections in children [4–6]. In a country with a high HIV prevalence among pregnant women, ranging from 30% to 50% in some areas [7], the effectiveness of an integrated PMTCT cascade has influenced other services, especially those available within ante-natal care (ANC) in all local health settings in SA. Although the impact of PMTCT integration has not been quantified in terms of health indicators and economic output, evaluations indicate that integration has helped to revitalise PHC [8, 9]. However, influence on other programmes in primary care has not included screening and managing non-communicable diseases (NCDs), like gestational diabetes mellitus (GDM), which are on the rise among pregnant women and their offspring in low- and middle-income countries like SA [10]. GDM is defined by the World Health Organisation (WHO) as “any degree of glucose intolerance with onset or first recognition during pregnancy” [11, 12]. It occurs in 2 to 9% of all pregnancies and increases pregnancy or delivery complications and long term risks of developing T2DM for both mothers and their babies, even though up to 95% of women with GDM revert to normal glucose levels after delivery [13–15]. GDM prevalence in SA is not exactly known but was estimated at 9.1% in a study conducted in 2018 [16]. Women who are diagnosed with GDM in SA are referred from their primary care clinics to receive ante-natal care and delivery services at the nearest tertiary facility. While those who are HIV positive return to their PHC facility for follow-up after delivery, there is no follow up intervention other than a referral letter for a six-week post-partum oral glucose tolerance test for women who had GDM, despite their well monitored ANC and delivery [17, 18]. Regardless of how other women’s health problems are treated, ART after delivery is consistently offered and monitored. Integrating GDM screening and T2DM prevention through PMTCT might be an innovative approach that could further improve health services for this same population (HIV positive women and their babies) and strengthen health systems in SA.

The PMTCT cascade begins with HCT, continues after delivery and has been universally integrated into ANC and postnatal care for both women and their exposed babies [19, 20]. Through PMTCT, many lessons have been learned in SA on how to adequately provide and maintain treatment for women during and after pregnancy—more so than for other people living with HIV (PLHIV) who are not as closely supported [1, 21]. Women under PMTCT learn to navigate the health system and to prioritise protecting their health post-partum. This reinforces their adherence to ART and leads to virtual elimination of mother-to-child transmission of HIV [21, 22] and could create opportunities for other linkages. In addition, due the high prevalence of HIV and increasing prevalence of GDM among pregnant women in SA [2, 23], the PMTCT integration experience offers opportunities and lessons for integrating GDM screening and T2DM prevention initiatives into existing PHC services.

This study aimed to assess PMTCT implementation outcomes, and how GDM screening and care and T2DM prevention were integrated into PMTCT in Western Cape Province (WC), SA. It also explored how the PMTCT experience might bridge gaps in screening GDM and preventing T2DM for women and their exposed babies at the primary level of care in SA.

### Study framework

This study framework draws from the WHO “six building blocks” model [24], as well as the health systems integration framework proposed by Atun et al. [25, 26]. In the latter, a “levels of integration” framework, analysis of interactions between programmes and interventions in health systems facilitates the determination and understanding of different integration levels. Possible levels of integration include **no integration**, when there is no formal interaction between programmes, **partial integration** ranging from (1) ***linkage***, or unstructured interactions, to (2) ***coordination*** with a committee to oversee their goal-oriented interchanges, but keeping separate structures, and finally to **full integration,** in which two programmes are merged in both their structural (funding, human resources, information systems) and functional elements (strategic planning, resource allocation, intervention delivery) [25, 27].

The six WHO health system building blocks [24] that contribute to the strengthening of health systems, in conjunction with health service integration are: 1) leadership and governance, 2) health information systems, 3) health financing, 4) human resources for health, 5) essential medical products and technologies, and 6) service delivery. Once access, coverage, quality and safety are ensured, improved health (in terms of level and equity), responsiveness, social, and financial risk protection and improved efficiency would follow.

The contexts in which health interventions are implemented differ and are likely to pose diverse facilitators and barriers to the process of integration. It is therefore important to bring together both key structural components of health systems and the processes related to interaction and integration of health programmes in a given context in order to arrive at a framework for analyzing and implementing integrated services.

**Fig 1:**
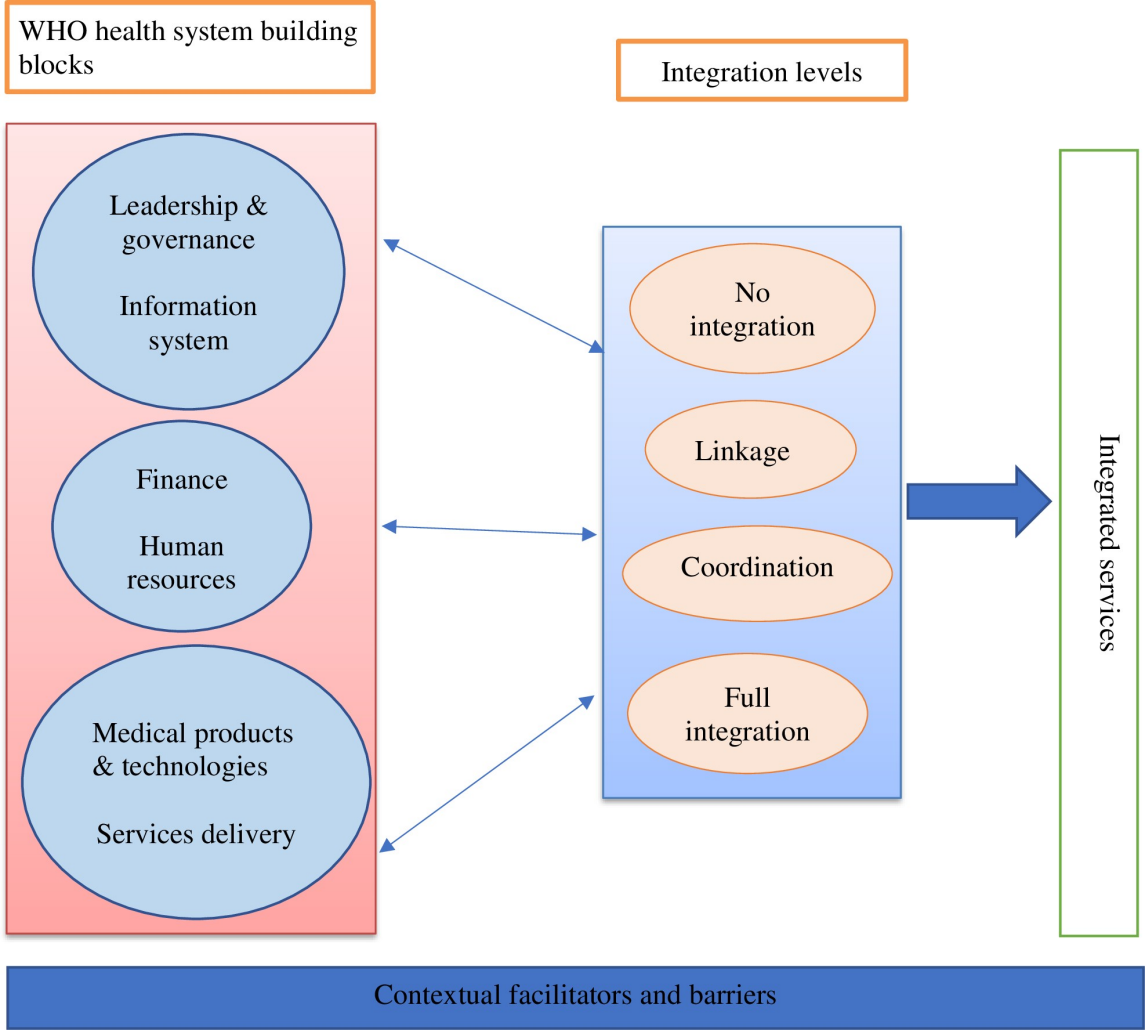
WHO building blocks and integration levels toward integrated services.

While our adapted framework included “no integration” and “full integration” as theoretical possibilities for eventual integration of GDM and T2DM management with PMTCT services, earlier formative research suggested that there was openness to and perceived feasibility of partial integration among health system actors [17]. The study therefore concentrated on this middle option. The adapted framework [24–27], proposed for this study is outlined in **Fig 1**.

## Methods

### Study design, setting and participants

A mixed method research strategy with a convergence design and data source triangulation was used [28, 29]. Having diversified data sets collected from various sources helped to strengthen our internal validity through triangulation [30]. This study contributes to and draws on an ongoing complex intervention implementation research project, IINDIAGO, aiming to integrate improved post-partum follow up for women with GDM into PHC and thus to contribute to prevention of T2DM in women with GDM in two South African provinces (WC–focus on Cape Town, and Gauteng–focus on Soweto township in Johannesburg). The overall research process was checked against the methodological quality criteria of the Mixed Methods Appraisal Tool (MMAT), version 2018 [31]. The methods and data sources included 1) documentary analysis of PMTCT and postnatal care policies; 2) semi-structured in-depth qualitative interviews with experts, health workers and women diagnosed with GDM while under PMTCT; and 3) quantitative longitudinal analysis of PMTCT implementation indicators across the WC for 2012–2017. A sub-analysis of outcomes for women with GDM under PMTCT and included in the PMTCT database was planned.

With regards to qualitative interviews, three groups of respondents were interviewed. The first were experts involved in research, programme development or evaluation related to integrating PMTCT into primary care at both national and provincial levels. The second were health workers drawn from public PHC facilities in Cape Town, WC Province, in order to inform the Cape Town arm of the ongoing IINDIAGO intervention study. The third were women with both GDM and HIV who were followed in PMTCT services in Cape Town. Respondents for all three groups of participants for semi-structured interviews were purposively selected.

#### Qualitative data collection and analysis

Two policy documents; 1. *The Western Cape Consolidated Guidelines for HIV Treatment*: *Prevention of Mother-to-Child Transmission of HIV (PMTCT)*, *Children*, *Adolescents and Adults–Amended version* [32], *and 2*. *The Western Cape Postnatal Care Policy* [33], developed by the WC Department of Health (DOH) experts, on the basis of national and international guidelines, were obtained as public records [34]. Content analysis [35] of the two documents was conducted to understand the PMTCT and postnatal care practices in SA and provide contextual information relevant to interpreting qualitative and quantitative data [35].

Interviews:

Experts: Eligibility criteria included experience in research, programme development or evaluation related to integrating PMTCT into primary care at both national, provincial and international levels. Three of the 13 experts contacted by email were unable to attend the interview due to schedule constraints, leaving a final sample of 10.

Health workers: Clinic managers, PMTCT nurses and midwives in clinics under the DOH of the city of Cape Town or WC province, with experience in organising PMTCT and ANC services were included in the study. Two of 12 health workers contacted by introductory email and a follow-up telephone were not available due to conflicting agendas and urgent matters to attend to in their facilities.

Patients: HIV+ women, aged 18 years and above, who were diagnosed with GDM while participating in the PMTCT programme during their ANC, and who delivered at a tertiary facility, were included. These women were interviewed about their satisfaction with PMTCT integrated services, and their perspectives on the desirability, facilitators, and barriers to GDM screening and T2DM prevention at PHC level. Recruitment took place between June 2016 and September 2016 for health workers, and was extended up to October 2017 for experts, and up to May 2018 for participating women. A total of 30 participants (n = 30) was reached for this study. All participants spoke English, but women who could not easily express themselves in that language were invited to take the interview in Xhosa through a fluent research assistant. Participants from the “patients” category were reimbursed for travel costs and received a 100 Rand (around 7 US dollars) voucher for appreciation to buy food or airtime, upon completion of the interview.

Interviews took place at the workplace or any other quiet setting suggested and agreed upon by both the interviewer and interviewee. Interview guides developed and tested for each category of participants were used, notes were taken during every interview, and all interviews were audio-recorded and transcribed. Interviews were conducted by a trained researcher (JCM) under the supervision of experienced qualitative researchers (KM and CZ). The researcher (JCM) introducing himself as a doctoral student and briefly interacted with the participants about the study before commencing the interviews. Each interview lasted between 45 and 90 minutes and interviews were conducted until no new information was obtained. By the end of all planned interviews, thematic saturation was achieved within each interview, and for all three groups of study participants. A coding system was hierarchically developed by JCM in collaboration with CZ using an inductive/deductive approach, and all discrepancies in the coding process were resolved through discussions between these two investigators. Content analysis was used for policy document while thematic analysis was used for interviews [36, 37]. ATLAS.ti software was used to assist data analysis and management. Interpretation of data was further supported through IINDIAGO team discussions and in relation to other research findings of the project, some of which have already been published [17, 38]. Characteristics of participants were analyzed by JCM using SPSS version 24.0.

#### Quantitative data collection and analysis

Panel and spatial data were analyzed using the annual number of women tested for HIV and the proportion of HIV+ women among women screened and the annual number of deliveries registered within the framework of the PMTCT programme. The data used were annual series for each of the 25 subdistricts of the WC Province. For deliveries registered under the PMTCT programme, the data series covered the period 2012–2017 while for the other two series, data were available for the period 2014–2016. To analyze the trend over time, a multilevel modeling approach was used. For each panel, a two-level linear hierarchical model with random intercept and slope [39, 40] has been estimated. Level 1 captures the evolution over time of each panel dependent variable using a growth curve of equation *Y*_*ti*_ = *π*_*0i*_ + *π*_1i_*(*TIME*_*ti*_) + *ε*_*ti*_. Where i = 1……25, t = 0…2 (for the two panel dependent variable related to HIV testing) or t = 0…‥5 (if the panel dependent variable was the number of women who gave birth under the PMTCT programme). The variable *Y*_*ti*_ is the value of the dependent variable in subdistrict i at time t, *π*_*0i*_ is the initial value (TIME = 0) in subdistrict i, *π*_*1i*_ is the rate of growth per unit of time in subdistrict i and the term *ε*_*ti*_ represents the residue which captures the unexplained variation of the evolution over time in the subdistrict i (*ε*_*ti*_ ⁓N (0, σ_ε_^2^). The equations of the level 2 model materialize the variability of the intercept (*π*_*0i*_) and slope (*π*_*1i*_) due to the specifics of each subdistrict. The equations of level 2 which have been considered are indicated below: *π*_*0i*_ = *β*_*00*_ + *r*_*0i*_ et *π*_*1i*_ = *β*_*10*_ + *r*_*1i*_. In these equations, *β*_*00*_ represents the general average at the initial time, *r*_*0i*_ is the random effect which corresponds to the deviation from the initial value *π*_*0i*_ of each subdistrict to the general average *β*_*00*_ (*r*_*0i*_ ⁓N (0, σ_0_^2^), *β*_*10*_ is the average growth rate per time unit and *r*_*1i*_ is the random effect which corresponds to the difference between the growth rate in each subdistrict and the average growth rate *β*_*10*_ (*r*_*1i*_ ⁓N (0, σ_1_^2^). The final equation obtained by combining the equations of the two hierarchical levels is written *Y*_*ti*_ = *β*_*00*_ + *β*_10_*TIME_*ti*_ + *r*_*0i*_ + *r*_1i_*TIME_*ti*_ + *ε*_*ti*_. To make the distributions of the dependent variables approximately normal, a basic logarithmic transformation was performed. The models were estimated using Restricted maximum likelihood (REML), which is recommended for small samples [39, 40]. The unstructured option was chosen to specify variance-covariance structure of the random effects. In addition, spatial autocorrelation of annual growth rates between subdistricts was analysed using Moran I global index and Moran I local index [41–44]. The values of these two indices generally vary between -1 and 1. The spatial autocorrelation is all the stronger as these indices are close to 1 or -1. Positive values translate positive autocorrelation and negative values translate negative autocorrelation. To test the statistical significance of these two indices, the spatial weight matrix was determined from the inverse of the distances between the different geographic units [45]. Spatial data were obtained from GADM (https://gadm.org/data.html). All statistical analyses were carried out with the software STATA/SE 14.2.

#### Ethics approval and consent to participate in the study

Ethics approval was obtained from the Human Research Ethics Committee, Faculty of Health Sciences, University of Cape Town (HREC REF: 946/2014), SA; and Comité d’éthique de la recherche (an accredited Research Ethics Board) du Centre Hospitalier de l’Université de Montréal (CHUM), (2018–6690, 17.044—ID), Canada. Written informed consent was obtained from each participant prior to the interviews, and filed for safekeeping. The anonymity of all study participants has been maintained throughout this research process.

## Results

The 3 groups of participants included: 10 pregnant women with HIV under PMTCT, diagnosed with GDM, 10 experts and 10 health workers—3 (15%) clinic mangers, and 7 (85%) PMTCT nurses and midwives. **Table 1**shows the characteristics of experts and health workers who participated in this study.

**Table 1:** Characteristics of experts and Frontline health workers participants.

Participant characteristics	N (%)
**Participants category**	
Experts or Key informants	10 (50%)
FHWCs	
Clinic managers	3 (15%)
Nurses and midwives	7 (35%)
**Sex:**	
Female	16 (80%)
Male	4 (20%)
**Age mean and SD:**	
Key informants	49.8
FHCWs	40.1
Overall mean (SD)	44.9 (8.2)

FHCWs: Frontline health workers; SD: Standard deviation

The synthesised results from policy documents, interviews, and PMTCT data in the WC are presented under the following three main headings, each with sub-headings: 1) current revised PMTCT and PNC guidelines; 2) integration of PMTCT and navigating systems for women with HIV and GDM; and 3) collaboration initiatives to enhance integration of programmes and retention of women.

### 1. Current revised PMTCT and PNC guidelines

Health system leadership is expressed in part through health policies, their dissemination, and implementation, as demonstrated in this study framework [24–26]. Interviewed experts explained that the policy documents and guidelines analysed in this study were developed by a team of provincial DOH-approved experts in relevant domains, e.g., HIV/PMTCT, maternal and child health (MCH), and health systems and in relation to provincial DOH priorities and the social, epidemiological and health system context. They were formulated based on the South African national consolidated PMTCT guidelines, that have themselves been drawn from the WHO consolidated guidelines. From time to time, protocols were revised to adjust to changing local circumstances. After amending the guidelines for PMTCT in 2015, covering HIV care for children, adolescents, and adults [32], the WC Department of Health initiated the postnatal care policy in 2016. This policy covers the first 1000 days of life, which includes pregnancy and two years after delivery [33]. Both policy documents have called for comprehensive ANC, including HIV counseling and testing, treatment initiation, and postnatal follow-up, to ensure adherence to PMTCT services. To achieve that, an integrated approach was recommended in the guideline amendments, but there was no mention of how it could be structured and implemented. Screening and care of GDM and other important NCDs whose rates are rising in the same population were not covered in the basic antenatal care documents that focused on elimination of vertical transmission of HIV [46, 47]. In addition to HCT and ART for women during ANC and for women with their babies after delivery, only two HIV opportunistic diseases—tuberculosis and cryptococcal meningitis—were included in the PMTCT policy document. The many other possible HIV co-morbidities were not mentioned.

#### Detailed but less inclusive PNC policies

Although the PNC policy laid a foundation for integrated care at the provincial level–with guidance for improving wellbeing of mothers and their babies in post-partum and beyond, such as HIV, contraception, domestic violence, and alcohol, or drug use–it did not include major NCD conditions. The increasing NCDs among pregnant women, such as diabetes, hypertension, other cardio-vascular conditions, and cancers, with harmful consequences to women and their babies–did not appear at all in this important document, nor were their screenings or treatments.

#### Human resources and training to implement integrated PMTCT

The implementation of health policy into practice depends on the way that guidelines are disseminated to health services and settings, available human and other resources such as medical products and technologies, as well as underlying contextual facilitators and barriers. Both experts and health workers discussed the implementation of the guidelines analysed above, in terms of service delivery. Their perspectives and comments reflected their differing roles and positions and hence experiences with these guidelines and practices (see key findings and illustrative quotes in **Table 2**).

**Table 2:** Results from interviews with experts and health workers by themes and illustrative quotes.

Key findings	Quotes from experts and health workers
**Theme 1: Current revised PMTCT guidelines**	
**Human resources and training to implement integrated PMTCT**	*“The guidelines have been aligned and the Integrated Management of Child Illness (IMCI) guidelines now incorporate HIV*, *ARVs and PMTCT Guidelines*, *now referred to as the Child Health Guidelines*. *On the ground*, *at facility level*, *there is always integration…*. *At the beginning it was challenging and it is still challenging*, *particularly when we have to implement new guideline because we’ve got to go through a whole process of training and retraining*. *The trouble with the whole HIV and PMTCT field is that it evolves and guidelines change rapidly or consecutively in close succession*, *so we need to keep retraining groups of people”* (Expert 1).
	*“I think in clinics where there are enough NIMART trained nurses it’s going well*. *You know*, *we have to do regular in-service training every time the guidelines change*, *make sure they are using the registers properly*. *There’s a PMTCT coordinator at the sub-structure district level who goes around*, *looks at the registers and does top-up training*,*…”* (Expert 3).
	*“I send the staff to the trainings on PMTCT*, *on BANC*, *and so on*, *and I think the sisters [nurses] are well equipped*. *It is part of my role to see who is running short and you can see I have that training record there and I can see who’s not trained on this or that*, *and then when there’s a space*, *I know that and I have to send her on this type of training*. *Regarding NIMART training*, *we have the help of MSF*. *MSF have been helping us and whenever we really run short of trained nurses through NIMART*, *then MSF just arrange their training*, *even if it’s only one sister*. *They conduct a class of one nurse and then make a follow-up*. *They would come to make a follow-up to that trained staff and make sure that she’s competent on what she was trained on*,*…”* (Health care worker 1).
**Uneven PMTCT implementation and better WC quantitative outcomes**	*“We wanted every pregnant woman to have an HIV test*. *So that’s the first PMTCT success [to identify HIV positive pregnant women]*. *The second success is to get them into treatment*, *and the third success is the baby is not infected*. *I started… What I say is that*, *I started off my career seeing babies die in their hundreds because of HIV*, *and now when I see what we did in 15*, *16*, *years*, *babies are living*. *We’ve managed paediatric HIV*, *so I see it as the most beautiful thing in the world to see what this country has done…*. *But as you know*, *it’s still hard*, *because we still have more people with HIV than anywhere else in the world*, *and we still don’t even know who they all are; and we still have to find them and put them on to treatment…*‥, *in Kwazulu Natal*, *it’s just spiralling out of control*. *It’s not stopping*. *It’s the epicentre*. *It’s where the fire is and we don’t know how to put off that fire”* (Expert 4).
	*“I’m just thinking back*, *in general and for Cape Town in particular*, *it’s a bit different from that of the broader South Africa*. *We basically received global funding*, *and in 2006*, *we appointed quite a number of PMTCT coordinators and with the trained PMTCT registered professional nurses within the MOU and PHC settings*, *as a means* of offering integrated ANC, ART and TB screening *into the broader PHC platform*. *So*, *a lot work has been done to close the PMTCT implementation loopholes* (Health worker 2).
**Theme 2: Integrated PMTCT and navigating systems for GDM care**	
**Partial integration of PMTCT and challenges to access unintegrated services**	*“I don’t think it’s fully integrated yet*, *but I think we’ve come a long way since when we started……*.*We had a bit of a hiccup when the immunisation was not really integrated into the HIV testing programme*, *and I think that is probably the one place where we still have a little bit of verticalisation on the whole issue of infant testing*” (Expert 1).
	*“So*, *my main objective within the PMTCT scope is to identify the patient when they come in*. *So*, *all patients who are HIV positive*, *I or my colleague chat with them and get them to see the counsellors as well*. *We make sure that they always have medication*. *We try to educate them regarding any side-effects and the obvious things like safer sex and the importance of adherence*. *We try to see where we can help them practically…*. *If there is any TB*, *other co-morbidities*, *if they have HIV and cardiac disease*, *we obviously see a lot of GDM*. *If there are any of these issues*, *then we will refer them to other departments within the hospital”* (Health worker 3).
**Efforts to keep women into integrated PMTCT**	*“…*.*You know what*, *the last mile is the mile of stigma and discrimination*. *It’s how we address that*. *So what happens is that we discriminate against HIV infected people*, *even in pregnant women in the hospital*, *so when I’ve looked at treatment failures*, *so why did children get HIV infected*, *it’s all around stigma*. *So the woman hasn’t disclosed her status to her family and she didn’t take the drugs*, *so she affects her baby…*‥*”* (Expert 4).
	*“So*, *you know*, *we have families that chase their loved ones away*. *If you’re gay or a lesbian*, *we have families that beat you up*. *So*, *if we want to do true integration*, *you have to get rid of stigma and discrimination and shame*, *and the way one human being treats another human being*, *and so that’s our problem”* (Expert 4).
**Theme 3: Collaboration initiatives to enhance integration and retention of women**	
**NGOs engagement**	*“Mothers to Mothers services which is well supporting the mothers who are pregnant and those after delivery*, *so they work hand in hand with antenatal labour ward as well as Child Health for continuity of care*. *So it has improved a lot based on other NPOs (non-profit organisational staff) that assist us if we have a problem with… We can’t find person*, *a patient*, *then mothers trace them*, *or the NPOs do trace them”* (Health worker 4).
	*“…‥ Another example which I’m personally familiar with is MomConnect*, *started two years ago to try and get pregnant women hooked up to a mobile health messaging*, *and quite quickly we achieved a 60%*, *currently running at about 60%*, *60*, *70% of all pregnant women are being registered on the system”* (Expert 2).
**Continuity of care in a context of migration**	*“Some go back to the Eastern Cape*, *I’m making now an example*. *So you don’t know*, *do they really rock up there and think that must be the same*? *If we have a system in place where if she goes to Jo’burg*, *when they punch in*, *say for instance*, *that folder number*, *here everything comes up*. *I’m just talking just health-wise*, *not in everybody’s eyes*, *but health-wise”* (Health worker 5).
	*“It is a problem with follow ups so*, *patients do move around*, *and if you don’t have an identifier can you can use across the country then it is difficult to trace*. *So*, *we deal mostly with patients that do return for care*. *Those that gets lost to follow-up*, *it’s difficult to get hold of because you don’t know where she went*, *what happened to her”* (Expert 5).
**Possibility of integrating GDM and other NCDs**	*“So*, *things can be scaled up very quickly if there is leadership from the national department and a willingness*. *So*, *the PMTCT system is such that people do take new interventions and can run with them quickly*. *So*, *that is something you could bear in mind for this Gestational Diabetes if it is seen to be a priority”* (Expert 4).
	*“I guess in terms of success*, *we see it very clearly*, *in terms of the number of lives saved*. *I guess all recent statistics have been consistently suggesting that in South Africa*, *the early life mortality due to HIV has reduced significantly*, *and this is essentially a result of the significant reduction of mother to child transmission…*‥ *the framework of prevention of mother to child transmission is a good setting*, *for instance*, *to provide good antenatal care and also good postnatal care*, *is an example for you of integration”* (Expert 5).
	*“…Even if the laboratory attached to the HIV Clinic*, *or to the Postnatal Clinic*, *it should provide*, *not only for the HIV*, *but for the screening of the risk of other conditions”* (Expert 6).
**Liaising with the communities**	*“I will say that they are helpful*, *because we use them as the bridge just to get to the communities*, *because they go the extra mile*, *in this way that they go inside those houses; then they will go there and give talk… For instance*, *when I was working in the TB Room*, *the CHWs will go and check if the patient is taking the medication correctly; or sometimes they will help them to take the treatment from us to them*. *So*, *sometimes they even help us to check*, *how is the condition of the room where the patient is staying in*? *Was there sanitation*? *Was there running water*, *and all that*? *So*, *they go deeper into the community whereas we are here in the facility*. *So*, *we use them in those terms*, *and also when we’re looking for the crèches*, *they know where are the crèches in the community*, *and how we go about getting in touch with the principal of those crèches*? *So*, *we use them for that*. *So*, *we work hand in hand with them”* (Health worker 4).
	*“For our HIV programmes*, *we have clinic-based counsellors and CHWs who are key part to the services…*.*in past I remember some suggested*, *the diabetic patients can benefit on that notion of peers helping others as well and CHWs*. *That systems of and CHWs*, *they need to be train*, *they need to be paid*, *that’s my opinion…When they are trained and offer their services*, *they have to be paid”* (Expert 6).

Experts who researched PMTCT policies and their implementation–some of whom had played a role in their development–explained how guidelines were integrated, in part to cover some MCH areas. They reported that amendments made by the DOH to adapt health systems to changing health needs, and increasing rates of NCDs like GDM, brought about other challenges; e.g., the need for more resources in terms of personnel and their training, supplies and medicines. An expert from PMTCT leadership in the province insisted on the need for more trained nurses through nurse-initiated and managed antiretroviral therapy and other continuous training schemes to ensure a fully integrated PMTCT, before considering it as an example for integrating other interventions.

Nurses discussed their training needs and expressed their interest in more specific training sessions beyond PMTCT, using different strategies in cooperation with various actors within and outside health systems, so that they could cope with the rising health needs requiring integrated services in their local clinics. One clinic manager, who runs a facility that received the health department’s award for reaching zero mother-to-child transmission of HIV in 2018, praised the role of trained nurses in HCT, treatment initiation and follow-up to monitor HIV viral load and to encourage and ensure adherence to ART, as key components of the successful PMTCT integration into PHC in the country. Training sessions to reinforce knowledge and best practices on these elements have been conducted in coordination with Médecins Sans Frontières, one of the non-governmental organisations (NGOs) that supports local health facilities in their efforts to deliver integrated HIV services into PHC in SA.

Since interviewed women were not involved in PMTCT guideline development or implementation, they did not share anything about policy documents, beyond expressing their wish for integrated services within the PMTCT programme. They expressed their desire to avoid navigating different facilities when they are screened for GDM or any other condition while under the PMTCT programme. One participant listed three facilities she visited in her journey for care, after she was diagnosed with GDM:

*“I booked at KTC Gugulethu, then they transferred me to Mowbray, after Mowbray, I came here* [*Groote Schuur Hospital*]*”*. (Women under PMTCT with GDM, Gugulethu 1)

When asked whether she would prefer to have integrated services and follow-up for her and her baby at the local clinic, instead of navigating between her local facility and hospitals, she replied with these words:

*“I know, it’s for me, I know I am just going to get that medication for me, I must also go and take medication for my baby; because sometimes you don’t go to the same clinic or we don’t have the same appointments….It would be easier for me if it’s the same clinic, and that is according to distance and people I am used to…”*. (Women under PMTCT with GDM, Gugulethu 1)

#### Uneven PMTCT implementation and better WC quantitative outcomes

Even though the scale-up of PMTCT within PHC was carried out in all South African provinces, facilitators and barriers were not the same everywhere, and hence there have been different outcomes [48, 49]. This situation is similar for GDM-related and other NCD services. Most provinces generally succeeded to reduce and control vertical transmission but some, and regions within provinces, still struggle in terms of some PMTCT indicators, due to historical health system inequities, and are considered a setback to the country’s overall good PMTCT performance [50, 51]. The WC province is one of the highest-performing provinces in SA across most health care indexes, especially HIV-related indicators [52–54]. The WC data revealed significant positive outcomes over the period of implementation of the PMTCT policies and guidelines since its initial rollout in 2002 but especially with the 2015 guidelines. The WC hosted the two first PMTCT trial sites in the country (pilot sites in Khayelitsha) in 1999 [49, 55] and its Government has since been working with local authorities, teaching institutions and NGOs, to mobilise resources towards integration of PMTCT services into all local health facilities [55].

According to the results of the estimation of the growth model (**Table 3**), there was generally a significant annual increase of 17.8% (95% CI: 12.9% - 22.0%) in the number of women screened for HIV between 2014 and 2016. Moran’s I global autocorrelation index is close to 0 and is not statistically significant (I = -0.039, p = 0.953). Thus, there is no spatial correlation between the subdistricts regarding the average annual growth rate of the number of women tested for HIV between 2014 and 2016.

**Table 3:** Results of the estimation of hierarchical linear models with random intercept and slope.

Variables	Dependent variables					
	Natural logarithm of the number of women tested for HIV		Natural logarithm of the proportion of HIV+ women among women tested		Natural logarithm of the number of women delivering on the PMTCT program^╧^	
	Coefficient	p	Coefficient	p	Coefficient	p
	(95% CI)		(95% CI)		(95% CI)	
Time (in years)	**0.164**^**ǂ**^	**0.000**	-0.010^ǂ^	0.761	**0.031**^**ǂ**^	**0.016**
	**(0.121–0.207)**		(-0.073–0.054)		**(0.006–0.057)**	
Intercept	9.049	0.000	1.020	0.000	4.685	0.000
	(8.627–9.472)		(0.787–1.253)		(4.066–5.305)	
**Random-effects Parameters**	**Estimate (95% CI)**		**Estimate (95% CI)**		**Estimate (95% CI)**	
Between-subdistrict variance	1.154		0.324		2.378	
	(0.653–2.041)		(0.175–0.601)		(1.326–4.262)	
Slope variance	0.007		0.009		0.001	
	(0.002–0.020)		(0.001–0.064)		(0.000–0.008)	
Intercept-slope covariance	-0.023		-0.011		0.013	
	(-0.071–0.026)		(-0.052–0.030)		(-0.028–0.053)	
Within-subdistrict variance	0.010		0.034		0.045	
	(0.006–0.018)		(0.020–0.060)		(0.034–0.060)	

^╧^Data was not available for the Bitou subdistrict.

^ǂ^Since the dependent variable has been transformed using the natural logarithm, this value corresponds to annual percentage increase /decrease of (e^beta^-1)*100. So we have 17.83% for beta = 0.164; -1.00% for beta = -0.010 and 3.15% for beta = 0.031.

However, according to Moran’s I local spatial autocorrelation index, there is a negative spatial correlation between some of the subdistricts (**Table 4**).

**Table 4:** Moran’s I local spatial autocorrelation index.

Subdistrict	Indicators					
	Average annual growth rate (in %) of the number of women screened for HIV		Average annual growth rate of the proportion of HIV + women among women tested		Average annual growth rate of the number of women delivering on the PMTCT program	
	I	p-value	I	p-value	I	p-value
**Beaufort West**	**-0.435**	**0.000**	-0.023	0.873	0.087	0.341
Bergrivier	-0.008	0.826	0.003	0.774	-0.017	0.873
Bitou	0.030	0.744	-0.020	0.922	//	//
Breede Valley	-0.040	0.988	-0.008	0.808	-0.013	0.842
Cape Agulhas	-0.037	0.973	0.127	0.211	0.075	0.426
Cederberg	-0.002	0.758	0.004	0.738	-0.067	0.873
City of Cape Town	-0.153	0.537	0.012	0.768	0.074	0.527
Drakenstein	-0.054	0.937	-0.006	0.826	0.024	0.693
George	0.207	0.281	0.003	0.844	-0.068	0.917
Hessequa	0.009	0.688	-0.074	0.805	-0.100	0.701
**Kannaland**	0.004	0.710	-0.009	0.801	**-0.518**	**0.001**
Knysna	0.229	0.268	0.001	0.861	-0.000	0.858
**Laingsburg**	-0.211	0.093	**-0.470**	**0.000**	0.014	0.659
Langeberg	-0.001	0.765	0.009	0.721	0.026	0.654
Matzikama	-0.021	0.845	-0.034	0.946	0.020	0.635
Mossel Bay	-0.113	0.606	-0.036	0.966	-0.043	0.997
Oudtshoorn	0.054	0.523	0.007	0.750	-0.143	0.555
Overstrand	0.010	0.758	0.017	0.728	0.128	0.332
Prince Albert	-0.098	0.664	0.006	0.726	0.132	0.252
Saldanha Bay	-0.005	0.821	-0.020	0.894	-0.029	0.934
Stellenbosch	-0.183	0.433	-0.169	0.484	0.105	0.424
**Swartland**	0.007	0.758	-0.020	0.890	**-0.412**	**0.028**
Swellendam	-0.070	0.820	0.132	0.187	-0.004	0.789
Theewaterskloof	-0.052	0.948	0.026	0.677	0.085	0.450
Witzenberg	-0.048	0.952	-0.111	0.539	-0.019	0.853

// Data not available

**Fig 2:**
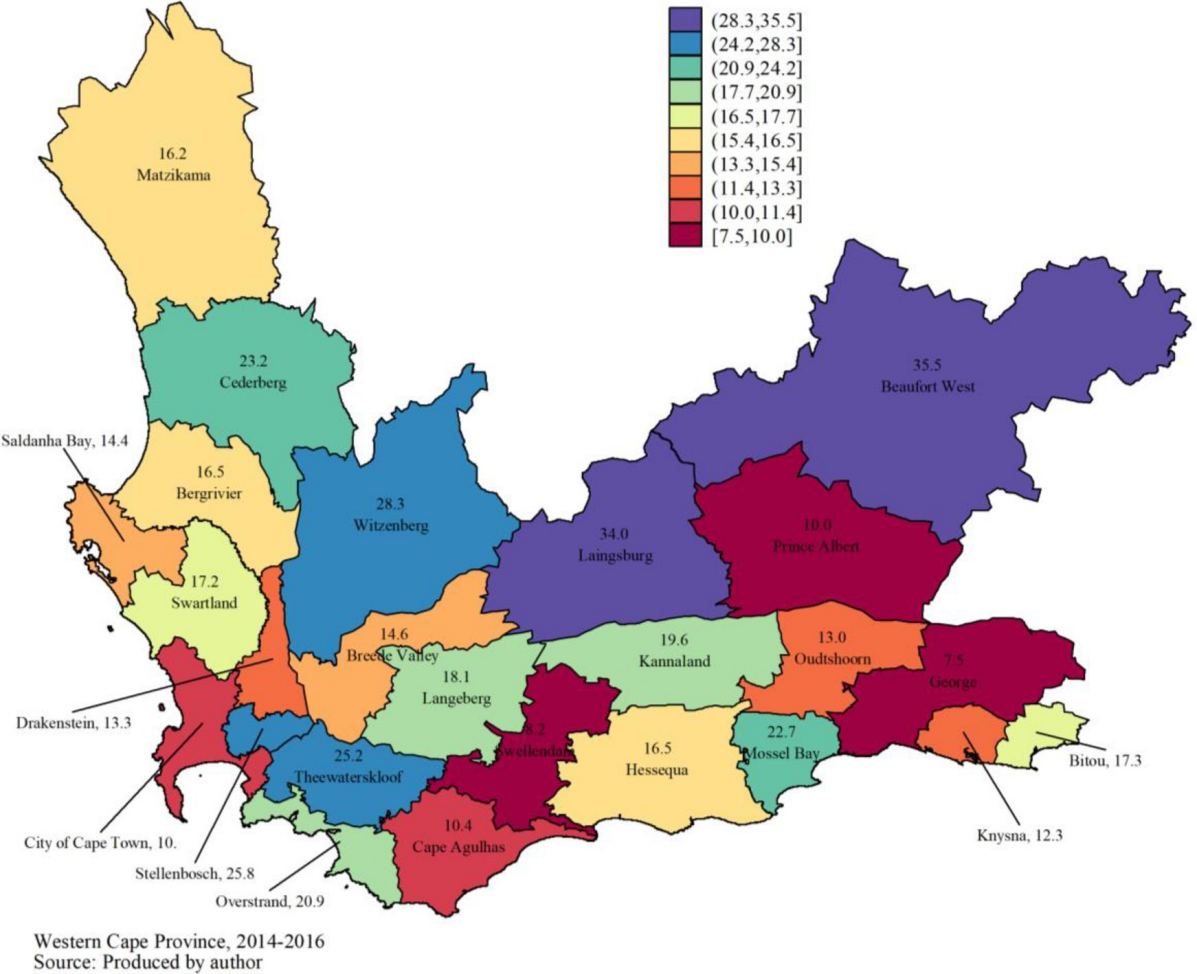
Average annual growth rate (in %) of the number of women screened for HIV between 2014 and 2016.

Indeed, the average annual growth rate of the number of women tested for HIV is relatively higher in one subdistrict (Beaufort West) compared to many neighboring subdistricts (**Fig 2**).

**Fig 3:**
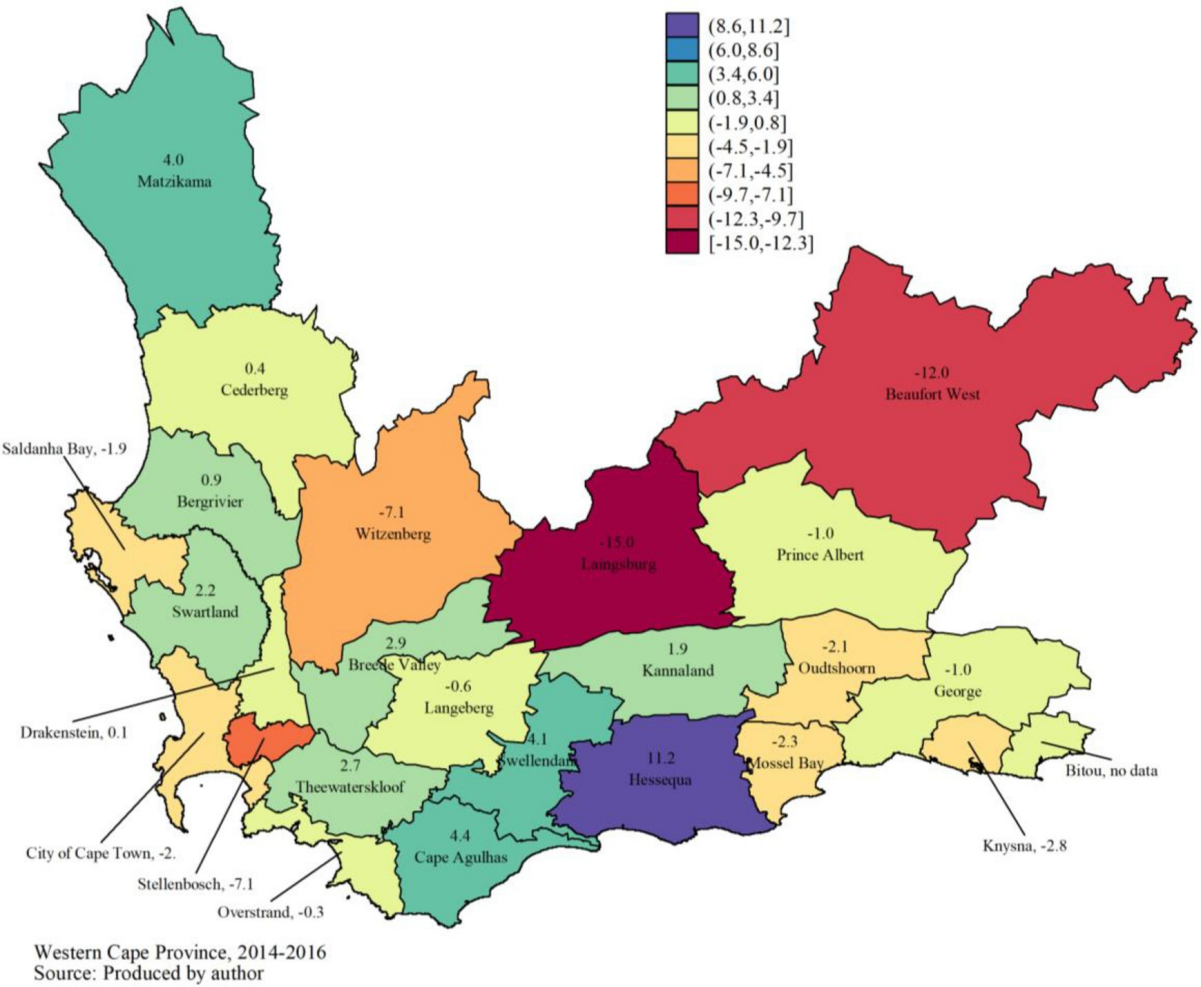
Average annual growth rate (in %) of the proportion of HIV + women among women tested between 2014 and 2016.

Regarding the proportion of HIV + women among women tested, on average, a non-statistically significant annual decrease of 1% (95% CI: -7.0% - 5.5%) was recorded (**Table 3**). Furthermore, the data do not show a partial correlation overall (I = -0.026, p = 0.687). However, a negative spatial correlation between the Laingsburg subdistrict and neighboring subdistricts has been highlighted (I = -0.470, p <0.001). This subdistrict recorded a significant drop in the proportion of women testing HIV positive compared to neighboring subdistricts (**Fig 3**).

**Fig 4:**
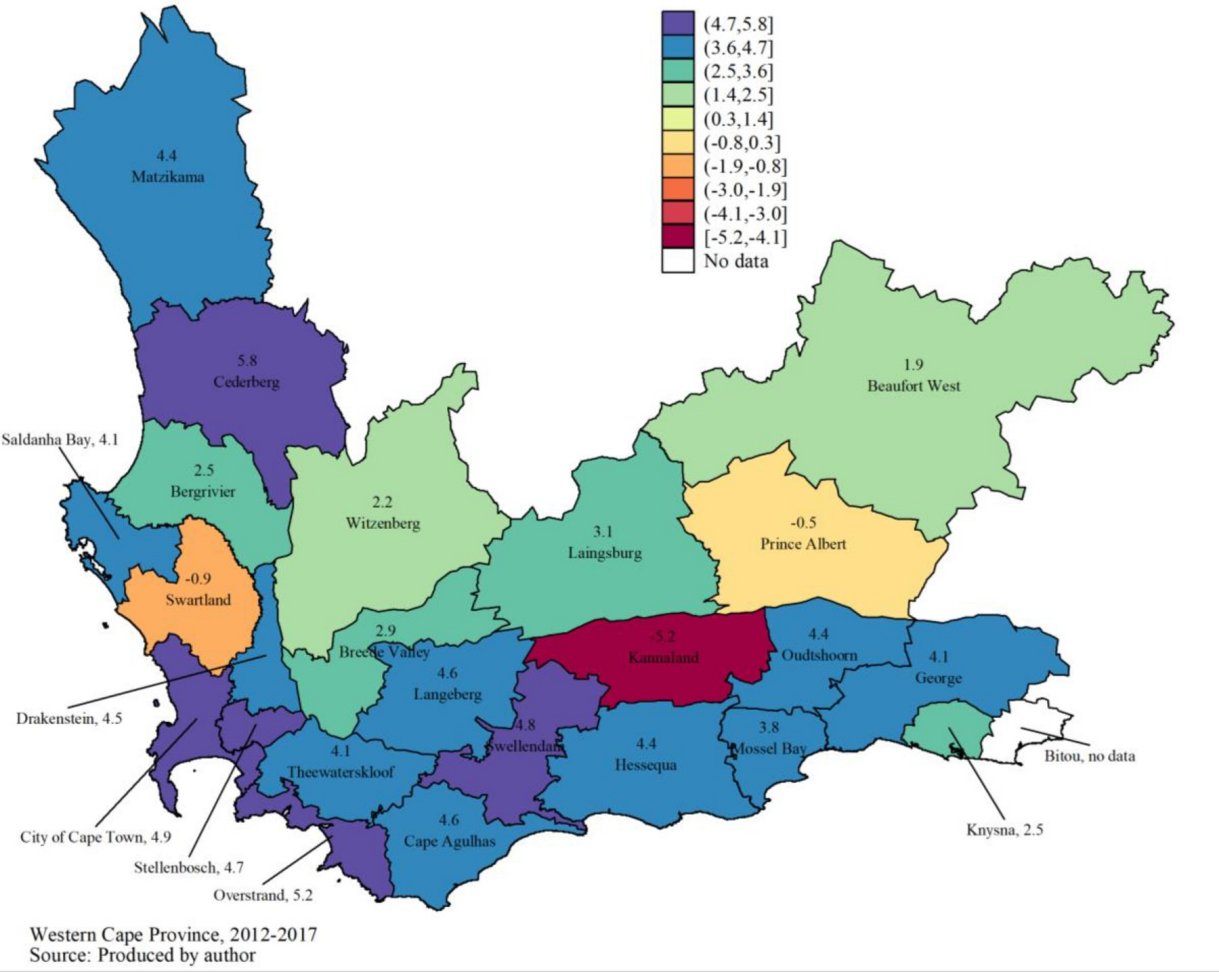
Average annual growth rate of the number of women delivering on the PMTCT program between 2012 and 2017.

The results of the growth model estimate recorded in **Table 3**indicate that there was generally a significant annual increase of 3.1% (95% CI: 0.6% - 5.9%) in the number of births recorded within the PMTCT programme between 2012 and 2017. Moran’s I global autocorrelation index is close to 0 and is not statistically significant (I = -0.028, p = 0.659). So, there is no spatial correlation between the subdistricts regarding the average annual annual growth rate of the number of women delivering on the PMTCT programme between 2012 and 2017. However, according to Moran’s I local spatial autocorrelation index (**Table 4**), there is a negative spatial correlation between the Kannaland subdistrict (I = -0.518, p = 0.001), Swartland subdistrict (I = -0.412, p = 0.028) and their neighboring subdistricts. Compared to neighboring districts, a decrease in the number of women who gave birth under the PMTCT programme was observed in these two districts (**Fig 4**).

No data were available about women in PMTCT who also had GDM.

### 2. Integration of PMTCT and navigating systems for women with HIV and GDM

Experts and health workers discussed different aspects of PMTCT integration into existing PHC services. Both agreed that PMTCT was partially integrated, which means that linkages and coordination were created between programmes and services available in every facility, as discussed in the study framework [24–26].

#### Partial integration of PMTCT and challenges to access unintegrated services

Although PMTCT results were praised with respect to integration into PHC services by all study participants, women who were diagnosed with GDM while under PMTCT said that they have struggled in their pursuit of care. In addition to their PMTCT programme, they were also required to visit tertiary health care facilities for pregnancy follow-up and delivery, because of their GDM diagnosis. Since the guidelines to screen and address such diseases within PMTCT do not exist, the women who were diagnosed with GDM while enrolled into PMTCT face barriers vis-à-vis transportation and safety issues, while going to the hospital for GDM follow-up. Taking medicines for both health problems, and exploring lifestyle changes are other challenges. Six out of ten women who continued their ART in the PMTCT programme at their clinics, wished to have their NCDs screened and managed within PMTCT at their local health settings. These women also spoke of the challenges in adhering to treatment and recommended lifestyle modifications, including challenges such as work stoppage with concurrent loss of income, and increased expenses for healthy food or a changing diet. However, one of the most inconveniencing experiences reported by these women was their separation from their usual local health care team while attending tertiary health facilities for their GDM care. One woman shared how she missed the PMTCT health team she was used to at her local clinic, while attending diabetic clinic at tertiary hospital:

*“…*.*That doctor*, *that is why I said he is like our father*, *you see*? *When you come to his room*, *when you go out you just have that hope… You can’t explain it*, *but you just have that hope…*, *I can’t lie*, *even the nurses there they know how to treat us*. *They don’t shout out*, *they just tell us we have to do this*, *even if you tell them the truth*, *the way you eat*. *They call the dietician to guide you*,*…”* (Women under PMTCT with GDM, Langa 7).

After a couple of visits to tertiary hospitals (Groote Schuur Hospital (GSH) and Tygerberg Hospital in the Cape Town metropolitan context), however, women reported that they adjust their routines and come to appreciate and recommend the high quality of services they receive there. One of the women who attended GSH for her GDM while under PMTCT explained:

*“I was scared to ask them* [*nurses*] *and because they were talking and treating us*, *I thought that this is the special hospital*. *Then I did ask the doctor and he said that this is the special hospital and that they are very good to treating Diabetes…*‥*That’s what I experienced here* [*GSH*]. *Now*, *when someone is pregnant*, *I tell them that if you will be treated in GSH*, *you will be fine”* (Women under PMTCT with GDM, KTC)

#### Efforts to keep women in integrated PMTCT

Despite their commitment to navigate a complex and fragmented health system in order to protect their own and especially their baby’s health during pregnancy, the issues raised above can cause retention problems in the long run, as some women default the programme or fail to take their regular medication. This is the reason why the DOH has recently requested that the lower level facilities also hand over their PMTCT services to the tertiary hospital for all referred GDM patients. This approach helped to fully integrate care for all co-morbidities during the pregnancy at well-equipped tertiary hospitals. GSH and Tygerberg hospitals have all the required critical services for mothers and their babies during pregnancy, during labour, and after delivery. They have trained nurses and midwives to care for these HIV positive individuals with GDM, and other co-morbidities. After delivery, these women go back to their local clinics for their ART and early infant diagnosis and immunisation services for their babies [3]. However, there are no follow-up initiatives to prevent or delay T2DM for women with a previous GDM diagnosis, and the interviewed health workers said their role ends with the PMTCT cascade, and PNC packages that do not include NCDs. PMTCT coordinators were assigned at each sub-district level in the province to ensure that everything regarding PMTCT is implemented, monitored and evaluated. However, their mandate is solely for PMTCT services, and this stance shows how partial its integration has been. All ten experts emphasised the successful results, and various benefits of partially integrated PMTCT into PHC throughout the country, and suggested approaches to move it to the next stage: full integration. They also discussed the journey that lies ahead, before the complete success of PMTCT programme could be achieved. They explained that discrimination and stigma became obstacles to the integration process because of psycho-social and health effects, with consequences to individuals, families and health care systems. While highlighting most of the persisting barriers like bureaucracy, poor management, lack of sufficient infrastructure and other resources at local facilities and provincial, or national health systems, 7 out of 10 experts firmly concluded that PMTCT has not been fully integrated and that this has complicated retention of these women. They based this conclusion on the failure to consider and include some MCH programmes that are very important for both women and their babies such as GDM screening and immunisation.

### 3. Collaboration initiatives to enhance integration and retention of women

Both experts and health workers who participated in this study discussed different collaboration initiatives and the underlying facilitators and barriers that led to PMTCT integration into existing PHC services. For cooperation efforts towards integrated PMTCT in policies and practice, various components from WHO building blocks and integration levels were mobilised, as discussed in the study framework [24–26].

#### NGO engagement

Integrated care of PMTCT and other health conditions that women face during pregnancy could not be achieved without the collaboration of different actors in health systems, NGOs and in communities. Experts and health workers stressed that NGOs such as Momconnect, Mothers to Mothers, and Médecins Sans Frontières have made substantial contributions to PMTCT implementation, and that this is reflected in the tangible results documented for PMTCT services. These organisations actively participated in key activities essential to the success of this programme, activities such as health education, home visits and sometimes intervening at the facilities, accomplished by trained members of the community who are supervised and incentivised by professionals working for these NGOs. These collaboration initiatives have been supplementing Governmental efforts to enhance HCT, initiate ART, keep women on treatment and trace the defaulters and all participants appreciated their roles.

#### Continuity of care in a context of migration

Tracing all women who default from treatment has been consistently difficult, as some had moved out of the province and could not be reached at all. Some women from other provinces come to work in Cape Town and book for PMTCT and ANC follow-up in the local facilities in Cape Town’s informal settlements. Some among them leave before or after delivery, returning mostly to the neighbouring Eastern Cape Province, but also elsewhere outside of the provincial boundaries. Experts and health workers recommended a nation-wide health information system, with unique identifiers that could help to trace women or other patients, wherever they book. With a nationally interconnected health system, women with co-morbidities could attend any health facility, have their health problems addressed and their medical reports stored and accessible for further visits or interventions elsewhere. Women who were interviewed in this study and who themselves navigated health systems for testing and treatment for multiple conditions could not offer any insights on this issue and have been relying on “Road to Health” booklets or transfer letters. Without health information system managing and transmitting electronic records at national level, some patients like these women may be lost or forgotten and when they migrate or attend a different facility, their diagnoses and medications are not known and this hinders the continuity of care. The use of unique identifiers through functioning health information systems could be a feasible and sustainable solution to this situation, as reported by experts who participate in this study.

#### Possibility of integrating GDM and other NCDs

Experts said that increasing the number of women benefiting from integrated HCT and delivering under PMTCT should not only be seen as a success story, but also as a window of opportunity for universal GDM or NCD screening, and subsequently initiating preventive measures for T2DM for both women and their babies. Most experts (80%) and clinic staff (70%) agreed on the feasibility of GDM and T2D integration but, unfortunately, without a clear policy and proper guidelines to include them into PHC based services like PMTCT, implementation of that integration is not likely possible in the current national and provincial context. According to the study respondents, prioritising health problems of such magnitude to this particular population, by authorities in national, provincial and municipal health departments, has to be the first step in the process of integrating them. Through PMTCT services, good results have been obtained and most of the interviewed experts asserted that it would be the logical and the right thing to do to integrate GDM screening and T2DM prevention measures with this important programme for women and their babies.

#### Liaising with the communities

The PMTCT cascade has the opportunity to integrate not only with other health services, but also within communities themselves. The existence of a well-functioning network within communities, and the trust that PMTCT has built over time, especially in liaising with the community health workers (CHWs) to follow-up with women, their babies, and their families, present an opportunity for screening and care of other health problems. The extensive role of CHWs has been recognized by each of the groups interviewed for this study: experts, health workers and women with GDM under PMTCT. They all emphasized the many important accomplishments health services integration achieved by CHWs, at clinics, and in communities. Before being transferred to the tertiary hospital for follow-up and delivery, one woman diagnosed with GDM while in the PMTCT programme at her clinic was asked about CHWs and their role in her pursuit of care. She immediately replied as follows:

*“I even have their cell numbers”*. She continued to say that CHWs assist women and their families in every situation, and ensure they attend the clinics or hospitals for regular administration of medication and follow-ups.

*“So, if maybe your date is tomorrow, they have to Whatsapp you, don’t forget about tomorrow”*.

She then elaborated, when asked whether CHWs only remind women about their appointments:

*“No*, *they’re dealing with the child that’s going to that clinic*. *They just remind us about our dates*. *Sometimes*, *they visit*. *Most of the time the people there have tuberculosis*. *Ja*, *they are good*. *They are doing good jobs*, *you see*, *sometimes if someone does not have the power to wash themselves*, *you know those people are very sick*, *they help and wash them or just to give them their breakfast”*. (Women under PMTCT with GDM, Langa 20).

Health workers interact with CHWs at the clinics, especially when there are many patients and queues to manage or when they are needed to give health talks, and their help is routinely sought to liaise with patients in the communities. All health workers agreed that CHWs are the key to successful interactions with women and other patients in the community. Experts praised the CHWs and added their voices to those of the health workers regarding the importance of the CHWs, adding that the CHWs could be more efficiently used to improve the overall PHC services. Experts noted that CHWs have played a critical role in other countries like Ethiopia, Rwanda and others and that they not only help at the clinics but also in assisting to deal with health problems of individuals and families in the communities. Experts encouraged offering training, supervision and payment to CHWs. This would motivate and give them the sense of responsibility, equip them with sufficient knowledge and, in fact help to resolve occasional cases of ethical concerns such as breach of confidentiality, issues regarding privacy and informed consent among others, as raised by some nurses closely working with them.

## Discussion

WHO’s “six building blocks” model and a health systems integration framework by Atun et al. [24–26], constituted the adapted framework for this study and each main result heading was conceptualised based on either or both components of this adapted framework. Components from the WHO building block model were used to review the current revised PMTCT and PNC guidelines, the health system integration framework was applied to the integration of PMTCT and navigating systems for women with HIV and GDM while components from both models were considered to elucidate the collaboration initiatives to enhance integration and retention of women.

The significant health and societal gains from PMTCT and extended HIV policies in SA have shown that an integrated approach can bridge structural gaps in service delivery across antenatal, intrapartum and postnatal care. PMTCT has been consistently implemented throughout the country, but concerns were raised by experts about the postnatal care that has not received the attention it is due, at both national and provincial levels, compared to antenatal and intrapartum care [56]. The question we have addressed was how PMTCT integrated into PHC is perceived by key stakeholders and reflected in policy and in empirical data, and specifically how women with both GDM and HIV are followed in PMTCT, in order to explore how the PMTCT experience might bridge gaps in screening GDM and preventing T2DM for women and their exposed babies at the primary care level in South Africa. Our hypotheses were two-fold: that the successful implementation and integration of PMTCT in the WC would offer lessons for integrating other important conditions, notably GDM; and that women with GDM would be reasonably well integrated into seamless care within the PMTCT programme because the lessons already learned about the value of PMTCT integration would have already been generalized to women with GDM. This study provides evidence in support of the first hypothesis, including widespread and strong support across stakeholder groups (experts, health workers and patients) for integration of GDM into PMTCT and into PHC. However, we found that women with GDM are poorly supported in integrated PMTCT–except if they are referred to have all of their ante-natal and delivery care at a tertiary facility.

PMTCT outcomes have been quantitatively assessed during the study period and the calculated number of women who attended HCT annually increased at 16.4%, while women who delivered under PMTCT increased at 3.1% annually in the WC, SA. These quantitative results corroborated the views of this study respondents regarding the integrated PMTCT services in the province and at national level.

Secondly, although there were no provincial data for women diagnosed with GDM while under PMTCT, the study participants suggested that the lessons from PMTCT should and can be extended, especially for interventions targeting the same population. This could apply beyond HIV care, notably in GDM and other NCD screenings and T2DM prevention, for which continuous testing, lifelong medication, behavioural change, and follow-up, as well as monitoring and evaluation would be needed. Integrating interventions like PMTCT into PHC has not been a simple or single step initiative, but rather a very complex intervention with multiple components. PMTCT has evolved from using a single dose of Nevirapine, to the combined regimens for eligible women [57], and to option B+ and to the PMTCT cascade extending from HCT, through pregnancy and delivery, post-partum care of mothers and infants, and lifelong ART for women. It has progressively been successfully integrated into PHC, due to enhanced training for nurses and closer monitoring and evaluation of services they provide. The positive assessments of the PMTCT programme by all categories of respondents as well as the quantitative data showing increased uptake of HCT, treatment initiation and subsequent long-term retention of women in ART, followed by reduction in HIV positivity rate, suggests that the integration approach can work well in the SA context. The experience has the potential to offer constructive ideas for planning other health interventions, especially those targeting the same population. Apart from a cohort study on selective screening strategies for Gestational Diabetes by Adam, S. and Rheeder, P. [58] in 2017, that included a limited assessment of HIV effect on the GDM incidence in the high HIV burdened South Africa, to our knowledge there is no other study that examined integrated GDM screening and subsequent follow-up for women under PMTCT. Our study suggests that GDM screening and T2DM prevention would ideally start with women who are already under PMTCT programme and then be expanded within PHC services.

Thirdly, GDM and T2DM have emerged as conditions increasingly affecting women in SA [16, 59], and their integration into this well-grounded PMTCT programme is seen as desirable, although with caveats. Dias et al in their recent study [60] highlighted that there are no compelling means to implement and integrate GDM screening and management into the existing MCH services and integrating it into PMTCT would therefore be one of the viable solutions to deal with it. PMTCT acceptability and adherence to treatment by women has generally increased over time, and has been the key to the success of the integrated PMTCT services in different contexts, despite pending socio-cultural, economic and leadership challenges [61–63], and would make integrated GDM screening and T2DM prevention feasible. As discussed in the results, women appreciate having the key health services they need in facilities that are accessible to their communities during pregnancy, and for their babies after delivery. Worries and fear expressed by women about GDM and the T2DM onset soon or later after delivery are similar to what they felt when tested positive to HIV and this could mean that they would abide to services for screening and treating GDM, and would initiate lifestyle changes to prevent T2DM, especially through its integration within PMTCT.

PMTCT owes its success, in part, to the supportive networks it enjoyed not only among donors and health systems but also from community actors whose contributions would also be relevant for other conditions affecting this same population, including GDM, T2DM and other NCDs. Working with community-based organisations has helped to empower HIV positive women, and reduced stigma and discrimination [64, 65] through providing information and counselling to patients and educating families and communities. The CHWs who are appreciated by all interviewed participants have emerged as an important force for supporting adherence, an important issue that surrounded PMTCT rollout [66, 67]. CHWs could under supervision contribute towards the integration of GDM screening and T2DM prevention into the PMTCT cascade, and other PHC services for the same population.

Finally, there are barriers to integrating the screening and care of GDM and prevention of T2D in SA, and as during PMTCT implementation, they could be progressively dealt with. From integrated GDM screening and follow-up during ANC, a period when HIV positive women are motivated to do whatever it takes to save their pregnancy and their babies [68], it could be feasible to retain them and their babies into postpartum care and follow-up. Using the PMTCT model of keeping previously pregnant women and their babies under care after delivery would bridge gaps within PNC, and follow-up with women who had GDM, along with their infants, for lifestyle change initiatives to prevent or delay T2DM onset and lessen the burden of tertiary care. This will however require changes in health policy, extra efforts in terms of training local health care providers, and attempting innovative approaches like integration in other well-implemented services, especially in public health facilities with limited resources [69, 70].

Without including the necessary screening programmes and care for GDM or subsequent T2DM prevention among other NCDs in the policy guidelines, a gap in practice would remain, and impact the results of PHC services. Prioritisation of GDM by the DOH and mobilisation of decision makers against this increasingly common chronic condition, that not only affect mothers, but also their babies, and their communities, is the key for its successful integration. However, integrating GDM management and initiatives to prevent or delay T2DM would add a significant layer of complexity to PHC. This goes beyond the relatively simple needs addressed through PMTCT during ANC, assessing viral loads and conducting PCR tests after delivery—it extends to teaching women to monitor their blood glucose levels, to modify their lifestyle and regularly visit the health facility to ensure their control is adequate or identify the need to refer for specialist care. Although this study provides a useful exemplar and protocol for future studies, there may be differences across the provinces and it may not be generalizable across SA.

## Conclusion

PMTCT programmes have not yet integrated the management of NCDs, notably GDM. However, there is significant learning from the PMTCT integration experience to suggest that GDM screening and care could be successfully integrated into PMTCT and PHC during ANC and initiate initiatives for preventing or delaying T2DM in postpartum. Key research is now needed in this area of integration to evaluate its effectiveness.

## Supporting information

S1 Checklist STROBE statement—checklist of items that should be included in reports of *cross-sectional studies*. (DOCX)

S1 File Mixed Methods Appraisal Tool (MMAT), version 2018. (DOC)

## Abbreviations

ANC: Ante-Natal Care
ART: Anti-Retroviral Therapy
CHWs: Community Health Workers
CI: Confidence Interval
DOH: Department of Health
GDM: Gestational Diabetes Mellitus
GSH: Groote Schuur Hospital
HCT: HIV Counselling and Testing
HIV: Human Immunodeficiency Virus
MCH: Maternal and Child Health
MMAT: Mixed Methods Appraisal Tool
NGOs: Non-Governmental Organisations
NCDs: Non-communicable diseases
PCR: Protein-Chain Reaction
PHC: Primary Health Care
PLHIV: People Living with HIV
PMTCT: Prevention of Mother-To-Child Transmission
PNC: Post Natal Care
REML: Restricted maximum likelihood
SA: South Africa
T2DM: Type 2 Diabetes Mellitus
WC: Western Cape
WHO: World Health Organisation
