# Supplementary material for: Integrating gestational diabetes and type 2 diabetes care into primary health care: Lessons from prevention of mother-to-child transmission of HIV in South Africa - A mixed methods study
Source: PLoS One. 2021 Jan 22;16(1):e0245229. doi: 10.1371/journal.pone.0245229 (PMC7822503; doi:10.1371/journal.pone.0245229)
Supplement: S1 File — (DOC) [file pone.0245229.s002.doc]

**Mixed Methods Appraisal Tool (MMAT), version 2018**

**Integrating gestational diabetes and type 2 diabetes care into primary health care: lessons from prevention of mother-to-child transmission of HIV in South Africa: - A mixed methods study**

| **Category of study designs** | **Methodological quality criteria** | **Responses** | | | |
| --- | --- | --- | --- | --- | --- |
|  |  | Yes | No | Can’t tell | Comments |
| Screening questions  (for all types) | S1. Are there clear research questions? | √ |  |  |  |
|  | S2. Do the collected data allow to address the research questions? | √ |  |  |  |
|  | *Further appraisal may not be feasible or appropriate when the answer is ‘No’ or ‘Can’t tell’ to one or both screening questions.* | | | | |
| 1. Qualitative | 1.1. Is the qualitative approach appropriate to answer the research question? | √ |  |  |  |
|  | 1.2. Are the qualitative data collection methods adequate to address the research question? | √ |  |  |  |
|  | 1.3. Are the findings adequately derived from the data? | √ |  |  |  |
|  | 1.4. Is the interpretation of results sufficiently substantiated by data? | √ |  |  |  |
|  | 1.5. Is there coherence between qualitative data sources, collection, analysis and interpretation? | √ |  |  |  |
| 2. Quantitative randomized controlled trials | 2.1. Is randomization appropriately performed? |  |  |  |  |
|  | 2.2. Are the groups comparable at baseline? |  |  |  |  |
|  | 2.3. Are there complete outcome data? |  |  |  |  |
|  | 2.4. Are outcome assessors blinded to the intervention provided? |  |  |  |  |
|  | 2.5 Did the participants adhere to the assigned intervention? |  |  |  |  |
| 3. Quantitative non-randomized | 3.1. Are the participants representative of the target population? |  |  |  |  |
|  | 3.2. Are measurements appropriate regarding both the outcome and intervention (or exposure)? |  |  |  |  |
|  | 3.3. Are there complete outcome data? |  |  |  |  |
|  | 3.4. Are the confounders accounted for in the design and analysis? |  |  |  |  |
|  | 3.5. During the study period, is the intervention administered (or exposure occurred) as intended? |  |  |  |  |
| 4. Quantitative descriptive | 4.1. Is the sampling strategy relevant to address the research question? | √ |  |  |  |
|  | 4.2. Is the sample representative of the target population? | √ |  |  |  |
|  | 4.3. Are the measurements appropriate? | √ |  |  |  |
|  | 4.4. Is the risk of nonresponse bias low? | - |  |  |  |
|  | 4.5. Is the statistical analysis appropriate to answer the research question? | √ |  |  |  |
| 5. Mixed methods | 5.1. Is there an adequate rationale for using a mixed methods design to address the research question? | √ |  |  |  |
|  | 5.2. Are the different components of the study effectively integrated to answer the research question? | √ |  |  |  |
|  | 5.3. Are the outputs of the integration of qualitative and quantitative components adequately interpreted? | √ |  |  |  |
|  | 5.4. Are divergences and inconsistencies between quantitative and qualitative results adequately addressed? | √ |  |  |  |
|  | 5.5. Do the different components of the study adhere to the quality criteria of each tradition of the methods involved? | √ |  |  |  |
